# The association between heatwaves and risk of hospitalization in Brazil: A nationwide time series study between 2000 and 2015

**DOI:** 10.1371/journal.pmed.1002753

**Published:** 2019-02-22

**Authors:** Qi Zhao, Shanshan Li, Micheline S. Z. S. Coelho, Paulo H. N. Saldiva, Kejia Hu, Rachel R. Huxley, Michael J. Abramson, Yuming Guo

**Affiliations:** 1 Department of Epidemiology and Preventive Medicine, School of Public Health and Preventive Medicine, Monash University, Melbourne, Australia; 2 Institute of Advanced Studies, University of São Paulo, São Paulo, Brazil; 3 Institute of Island and Coastal Ecosystems, Ocean College, Zhejiang University, Zhoushan, China; 4 College of Science, Health and Engineering, La Trobe University, Melbourne, Australia; Chinese University of Hong Kong, HONG KONG

## Abstract

**Background:**

To our knowledge, no study has assessed the association between heatwaves and risk of hospitalization and how it may change over time in Brazil. We quantified the heatwave–hospitalization association in Brazil during 2000–2015.

**Methods and findings:**

Daily data on hospitalization and temperature were collected from 1,814 cities (>78% of the national population) in the hottest five consecutive months during 2000–2015. Twelve types of heatwaves were defined with daily mean temperatures of ≥90th, 92.5th, 95th, or 97.5th percentiles of year-round temperature and durations of ≥2, 3, or 4 consecutive days. The city-specific association was estimated using a quasi-Poisson regression with constrained distributed lag model and then pooled at the national level using random-effect meta-analysis. Stratified analyses were performed by five regions, sex, 10 age groups, and nine cause categories. The temporal change in the heatwave–hospitalization association was assessed using a time-varying constrained distributed lag model. Of the 58,400,682 hospitalizations (59% women), 24%, 34%, 21%, and 19% of cases were aged <20, 20–39, 40–59, and ≥60 years, respectively. The city-specific year-round daily mean temperatures were 23.5 ± 2.8 °C on average, varying from 26.8 ± 1.8 °C for the 90th percentile to 28.0 ± 1.6 °C for the 97.5th percentile. We observed that the risk of hospitalization was most pronounced for heatwaves characterized by high daily temperatures and long durations across Brazil, except for the minimal association in the north (the hottest region). After controlling for temperature, the association remained for severe heatwaves in the south and southeast (cold regions). Children 0–9 years, the elderly ≥70 years, and admissions for perinatal conditions were most strongly associated with heatwaves. Over the study period, the strength of the heatwave–hospitalization association declined substantially in the south, while an apparent increase was observed in the southeast. The main limitations of this study included the lack of data on individual temperature exposure and measured air pollution.

**Conclusions:**

There are geographic, demographic, cause-specific, and temporal variations in the heatwave–hospitalization associations across the Brazilian population. Considering the projected increase in frequency, duration, and intensity of heatwaves, future strategies should be developed, such as building early warning systems, to reduce the health risk associated with heatwaves in Brazil.

## Abstract 2

Qi Zhao and colleagues reveal the association between heatwaves and hospital admissions between 2000 and 2015 in Brazil, with young children and the elderly most affected.

## Author summary

**Why was this study done?:**

- Brazil has a rapidly growing morbidity burden and is experiencing some of the most severe effects of global warming. However, there is no national evidence regarding the heatwave–hospitalization associations.

**What did the researchers do and find?:**

- Using a data set covering >78% of the national population (>160 million residents), we performed a nationwide time series study to examine the relationship between heatwaves and risk of all-cause and cause-specific hospitalizations and how it varied over time among the Brazilian population.
- Exposure to heatwaves characterized by high temperatures and long durations was associated with higher risk of hospitalization compared with moderate heatwaves. The duration of severe heatwaves was additively associated with risk of hospitalization in the cooler south and southeast regions.
- Children and the elderly were most vulnerable to heatwaves. Hospital admissions for perinatal conditions were most strongly associated with heatwaves. Over the study period, the risk of hospitalization associated with moderate heatwaves declined substantially in Brazil (especially in the south), with the notable exception of an apparent increase in the southeast region.

**What do these findings mean?:**

- To the best of our knowledge, this is the first national study to explore the geographic, demographic, cause-specific, and temporal variations in the association between heatwaves and risk of hospitalization in Brazil.
- Considering the projected increase in the frequency, duration, and intensity of heatwaves, future strategies for the forecasting of healthcare utilization in the Brazilian population should take into account the association between heatwaves and hospitalizations, especially among the most vulnerable population subgroups.
- Given the unique location of Brazil and its climatic and geographic diversity, our findings may be of relevance for other South American countries.

## Introduction

Globally, the average temperature has risen by 1.7 °C since 1910, accompanied by increased frequency, duration, and intensity of extreme heat events, otherwise known as heatwaves [1]. Heatwaves are among the most life-threatening meteorological factors, causing more excess morbidity and mortality than other natural disasters due to their large scope of influence and inadequate preparation [2]. In 2003, a heatwave across Europe caused more than 72,000 excess deaths in 14 European countries, while over 55,000 Russians are estimated to have lost their lives because of the sustained extreme heat in the summer of 2010 [3].

There is no unified criterion regarding the critical temperature threshold and duration of heatwaves worldwide. For example, in Sweden, a heatwave is defined as at least five adjacent days when the daily maximum temperatures are above 25 °C [4]. In Maine, United States, a heatwave is confirmed if there are more than two adjacent days with daily temperatures >35 °C [5]. However, most definitions of heatwaves are based on the total adverse effect on the population as a whole, without considering how the effect may vary across age and sex groups and among patients with pre-existing disease conditions [6].

South America is experiencing some of the most significant effects of global warming compared with many other areas of the world [7]. Brazil—the fifth most populous country—has more than 207 million people living in an area of land that covers nearly half of the South American continent [8,9]. Life expectancy in the Brazilian population has increased by more than 10 years since 1990, to 76 years in 2016 [10]. Consequently, the burden of morbidity has remained largely unchanged in the past quarter century, placing considerable pressure on its universal healthcare system [11]. Brazil has one of the world’s fastest-growing economies of recent decades [12]. Findings from high-income countries in the Northern Hemisphere have indicated that rapid economic development may confer some degree of protection against the adverse health outcomes associated with extreme heat [13,14]. Whether this is also true for populations from lower–middle-income countries in the Southern Hemisphere remains unknown.

Given the populous nature of Brazil and the vulnerability of the South American continent to climate change, we have started to document the growing impact of climate change on healthcare utilization in Brazil [15–17]. The climatic diversity and the unique disease burden of this country suggest that the association between heatwaves and health outcomes may differ from other countries [18,19]. In this study, we use a national data set to quantify the geographic, demographic, cause-specific, and temporal variations in the association between heatwaves and risk of all-cause and cause-specific hospitalizations in the Brazilian population.

## Methods

This time series study is reported as per the Strengthening the Reporting of Observational Studies in Epidemiology (STROBE) guidelines (S1 Text). The data analyses for the present study were performed following a prospective analysis plan (S2 Text). Changes in the analysis plan are described in S2 Text.

### Study area

There are five regions in Brazil—the north, northeast, central west, southeast, and south (S1 Fig). This geographic classification is defined officially by Brazil’s National Institute of Statistics (IBGE) according to the similarities of states and cities in climatic and socioeconomic characteristics. There are three main climatic zones in Brazil, including a tropical climate in the north and central west, a semiarid climate in the northeast, and a subtropical/temperate climate in the south and southeast [19]. The population density was lowest in the north (4 persons/km^2^) and highest in the southeast (87 persons/km^2^) [18,20]. Economically, the southeast is the most developed region, where the socioeconomic disparity is also significant: over half of Brazil’s slums or poor settlements are located in this region [20]. In contrast, the south has the most equitable level of socioeconomic development.

### Data collection

#### Hospitalization data

For each city, time series data on hospitalization between 1 January 2000 and 31 December 2015 were collected through Brazil’s National Unified Health System. There were 5,570 cities in Brazil at the end of 2015, but the electronic medical records of some cities were not completed during the early years. To reduce the impact of missing data, we only applied for hospitalization records in 1,814 cities that had complete data for the 16-year study period. The population coverage of our data set ranged from 26% in the north to 87% in the south (>78% at the national level). Medical variables included information on patient’s sex, age (0–4, 5–9, 10–19, 20–29, 30–39, 40–49, 50–59, 60–69, 70–79, or ≥80 years), admission date, city of residence, and primary diagnosis coded according to the International Classification of Diseases, 10th revision (ICD-10). The hospitalization data were then divided into nine main cause categories according to primary diagnosis (S1 Table).

#### Meteorological data

For each city, daily minimum and maximum temperatures were extracted from a 0.25° × 0.25° meteorological data set developed by Xavier and colleagues [21] using an inverse distance weighting interpolation with data from 735 weather stations. Specifically, data from the grid overlaying the center of each city were applied. Daily mean temperature was computed as the mean of minimum and maximum temperatures. In addition, data on daily relative humidity (RH) were obtained from 193 city-specific weather stations during 2000–2012 via Brazil’s National Institute of Meteorology.

### Definitions of heatwaves

Although without a unified definition, a heatwave for a specific area is commonly considered as an extreme heat event that persists for ≥2 days, compared with its long-term weather status [2]. Some heatwave studies applied various temperature indicators, including daily minimum, mean, and maximum temperatures [22,23]. However, findings from a recent multicountry study suggest that daily mean temperature may perform better for estimating the heatwave–health association than daily minimum and maximum temperatures [24]. In this study, we defined heatwaves using the daily mean temperature, as it represents the overall thermal condition of a day. In this study, we applied 12 heatwave definitions (S2 Table) by combining thresholds at the 90th, 92.5th, 95th, or 97.5th percentiles of city-specific year-round daily mean temperatures and durations of ≥2, 3, or 4 consecutive days, respectively. Compared with using unified temperature values as thresholds, these definitions were able to take into consideration the long-term local acclimatization [24,25]. Longer durations were not applied due to the extremely low frequency for days with high temperature thresholds.

### Statistical analyses

#### Heatwave–hospitalization association

Data analyses were conducted for the hottest five consecutive months in each city, which were identified using the city-specific monthly mean temperatures during 2000–2015. A two-stage approach was applied to quantify the heatwave–hospitalization association [24,26]. In the first stage, a quasi-Poisson regression with constrained distributed lag model was used to estimate the relative risk of hospitalization (with 95% confidence interval [CI]) in each city separately during heatwave days compared with nonheatwave days. For each city,

$$N_{j}\sim poisson\;(\mu_{j})$$

$$Log\;(\mu_{j})\;=\;\alpha +cb({HW}_{j},\;lag\;=\;7)+\beta {Stratum}_{j}+\gamma {Dow}_{j}+\delta {Holiday}_{j},$$ (1)

for which N_j_ is the daily hospitalization counts on day j. HW_j_ is a binary variable representing heatwave days (yes or no). cb(HW_j_, lag = 7) is the cross-basis function to fit the cumulative association on lag 0–7 days, with a natural cubic spline with 3 degrees of freedom (df) used for lag days. Stratum_j_ is a categorical variable of the year and calendar month (e.g., 2000-Jan, 2000-Feb, or 2000-Mar) to control for the within-season variation and long-term trend. In total, there were 192 strata (16 × 12). This design provides reliable control for temporal variations [27,28]. Dow_j_ is a categorical variable representing day of the week. Holiday_j_ is a binary variable representing public holidays in Brazil. α is the intercept. β, γ, and δ are coefficients.

In the second stage, city-specific estimates fitted by Eq 1 were pooled at the national level using a random-effect meta-analysis with maximum likelihood estimation [29]. Stratified analyses were performed by five regions, sex, 10 age groups, and nine main cause categories.

#### Added risk associated with the duration of heatwaves

Findings from previous studies have suggested that the association between heatwaves and mortality may be deconstructed into two parts [24]: (1) the independent health risk of daily high temperature and (2) the added health risk due to the duration of extreme heat. To examine whether the added health risk existed for Brazilian hospitalizations, the two-stage analysis was performed by controlling for daily mean temperatures. Our initial analysis using a distributed lag nonlinear model indicated that the association between daily mean temperature and hospitalization was linear (S2 Fig). Therefore, an additional cross-basis function was added into Eq 1, which combined a linear function for daily mean temperature and a 3-df natural cubic spline for the lag of temperature up to seven days.

#### Temporal variation in the association

A similar two-stage approach was applied to estimate the heatwave–hospitalization associations at two specific early and late time points of the study period—the middle days of the hottest five consecutive months in 2002 and 2013. This strategy has been described in detail by Gasparrini and colleagues [14] to explore whether there is temporal variation in the temperature–health association. In the first stage, the heatwave–hospitalization associations at the two time points in each city were estimated using the quasi-Poisson regression with time-varying constrained distributed lag model [14,30]. Specifically, assuming a linear temporal change, an additional linear interaction term was added into Eq 1, which multiplied cb(HW_j_, lag = 7) with a time variable centered at the specific time point in 2002 or 2013. In the second stage, the city-specific estimates were pooled at the national level using a random-effect meta-analysis with maximum likelihood estimation.

#### Sensitivity analyses

Sensitivity analyses were conducted to test whether our results were reliable by extending the lag from 0–7 days to 0–9 days and df of lag days from three to five. We evaluated the confounding effect of RH using observed data from 193 cities during 2000–2012.

R software (version 3.4.1) was used to perform all data analyses. The “dlnm” package was used to examine the distributed lag relationship between heatwaves and hospitalizations [31]. The “mvmeta” package was used to perform meta-analyses [32].

## Results

During the hottest five consecutive months between 2000 and 2015, there were 58,400,682 (59% women) hospitalization cases in the 1,814 Brazilian cities (Table 1). Specifically, 24%, 34%, 21%, and 19% of cases were aged <20, 20–39, 40–59, and ≥60 years, respectively (2% of cases without age information). The city-specific year-round daily mean temperatures were 23.5 ± 2.8 °C on average, varying from 26.8 ± 1.8 °C for the 90th percentile to 28.0 ± 1.6 °C for the 97.5th percentile (S3 Table). Regionally, the average daily mean temperature ranged from 20.2 ± 1.5 °C (25.3 ± 1.2 °C for the 90th percentile and 27.0 ± 1.1 °C for the 97.5th percentile) in the south to 27.1 ± 0.7 °C (28.7 ± 0.6 °C for the 90th percentile and 29.5 ± 0.6 °C for the 97.5th percentile) in the north. For all heatwave definitions, the average number of heatwave days was similar across the five Brazilian regions, declining from 26 days for 90th_2d to 3 days for 97.5th_4d per year.

**Table 1:** Hospitalizations (cases and crude rate) and annual heatwave days in 1,814 Brazilian cities by region.

	Cases (rate%)	Mean annual days (±SD) of 12 heatwave definitions for each city											
		90th (2d)	90th (3d)	90th (4d)	92.5th (2d)	92.5th (3d)	92.5th (4d)	95th (2d)	95th (3d)	95th (4d)	97.5th (2d)	97.5th (3d)	97.5th (4d)
**National**	58,400,682 (2.5)	26 ± 4	21 ± 4	18 ± 4	19 ± 4	15 ± 3	12 ± 3	12 ± 3	9 ± 2	7 ± 2	6 ± 2	4 ± 2	3 ± 1
**North**	1,604,910 (2.5)	28 ± 4	22 ± 4	18 ± 5	21 ± 2	16 ± 3	13 ± 3	14 ± 2	11 ± 2	8 ± 2	7 ± 1	5 ± 1	4 ± 1
**Northeast**	17,130,114 (2.7)	26 ± 5	21 ± 4	18 ± 4	19 ± 4	15 ± 3	12 ± 3	12 ± 3	9 ± 2	7 ± 2	5 ± 2	4 ± 1	3 ± 1
**Central west**	4,845,190 (2.8)	26 ± 3	21 ± 3	18 ± 4	20 ± 3	16 ± 3	13 ± 3	13 ± 2	10 ± 2	8 ± 2	6 ± 1	5 ± 1	3 ± 1
**Southeast**	25,230,159 (2.3)	24 ± 4	20 ± 4	16 ± 4	17 ± 3	14 ± 3	11 ± 3	11 ± 3	9 ± 2	7 ± 2	5 ± 2	4 ± 1	3 ± 1
**South**	9,590,309 (2.7)	28 ± 3	24 ± 3	20 ± 2	21 ± 2	17 ± 2	14 ± 2	14 ± 2	11 ± 2	9 ± 2	7 ± 1	5 ± 1	4 ± 1

**Fig 1:**
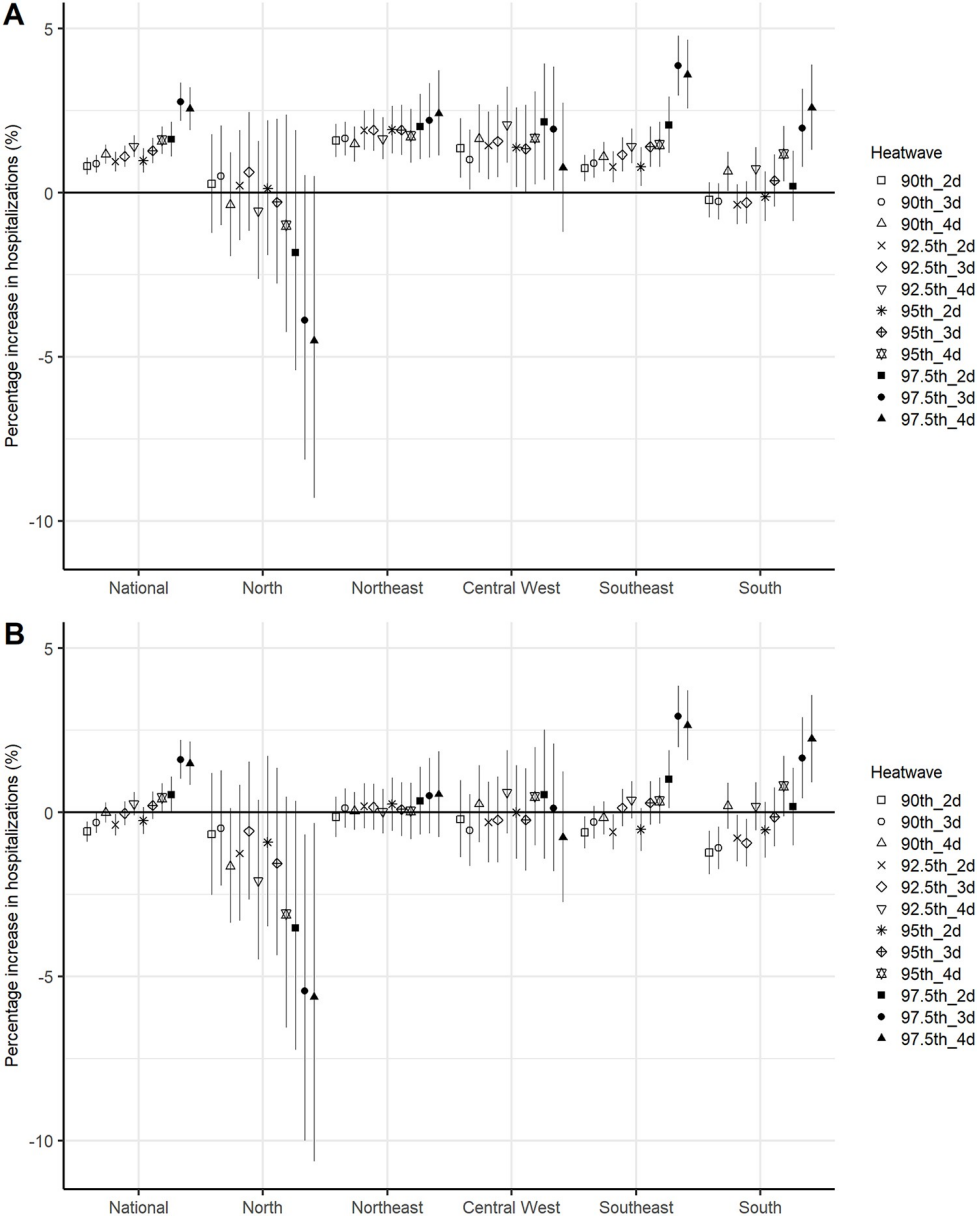
Associations between heatwaves (12 definitions) and risk of all-cause hospitalization by region. (A) Shows the results without controlling for daily mean temperature, representing the overall association between heatwaves and risk of hospitalization. (B) Shows the results after controlling for daily mean temperature, representing the added risk of hospitalization associated with heatwave duration.

At the national level, heatwaves characterized by high temperatures and long durations (i.e., severe heatwaves) were associated with higher risk of hospitalization than heatwaves with low temperatures and short durations (i.e., moderate heatwaves) over lag 0–7 days (Fig 1A). The risk of hospitalization increased from 0.8% (95% CI 0.6%–1.2%) over 90th_2d to 2.6% (95% CI 1.9%–3.2%) over 97.5th_4d, compared with nonheatwave days. The cumulative associations between heatwaves and risk of hospitalization were significant in the southeast, northeast, and central west and minimal in the north. In contrast, only heatwaves lasting ≥4 days were substantially associated with hospitalizations in the south. The associations between heatwaves and hospitalizations were greatest on the first day of exposure and were followed by hospitalization displacements after lag 3 days (S3 Fig). The heatwave–hospitalization relationship disappeared for most heatwave definitions after controlling for daily temperatures across the five Brazilian regions (Fig 1B). However, heatwaves with a threshold ≥97.5th percentile of year-round temperatures were still positively associated with hospitalizations in the southeast and south.

**Fig 2:**
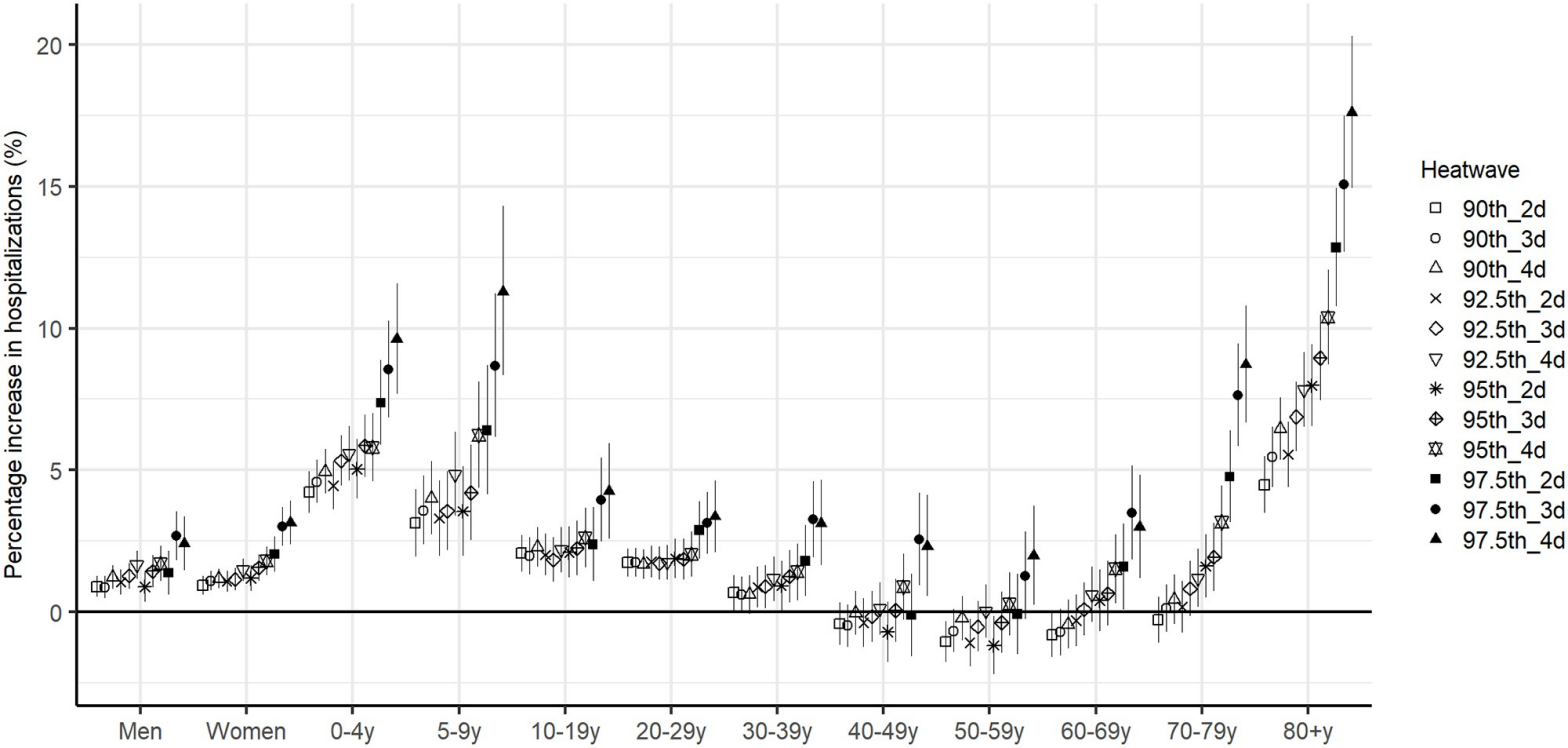
Associations between heatwaves (12 definitions) and risk of all-cause hospitalization by sex and age.

There was no substantial sex difference in the cumulative association between heatwaves and hospitalizations (lag 0–7 days), but it varied by age group (Fig 2). The heatwave–hospitalization relationships were strongest in children 0–9 years and the older population ≥70 years, particularly during severe heatwave days. For example, the risk of hospitalization increased by 18% in the elderly ≥80 years and 11% in children 5–9 years over 97.5th_4d. However, the subgroups 40–69 years were only substantially affected by severe heatwaves, such as 97.5th_3d and 97.5th_4d. The heatwave–hospitalization associations were greatest on the first day of exposure and declined thereafter in most population subgroups (S4 Fig).

**Fig 3:**
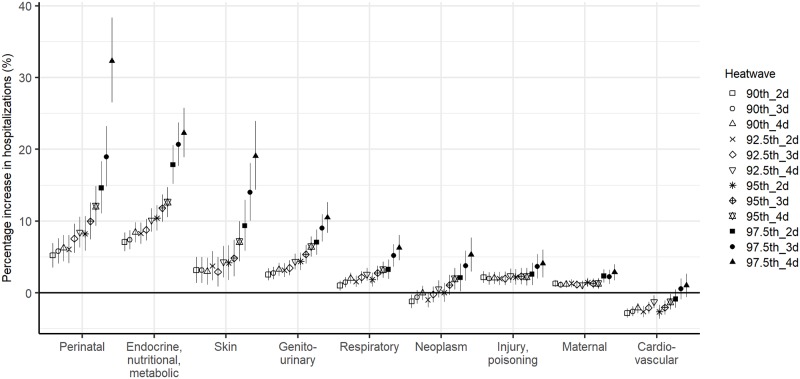
Associations between heatwaves (12 definitions) and risk of hospitalization for nine cause categories.

The heatwave–hospitalization associations varied across the nine cause categories (Fig 3). The strongest association (lag 0–7 days) occurred for hospitalizations for perinatal problems over 97.5th_4d. In addition, admissions for endocrine, nutritional and metabolic diseases, skin problems, and genitourinary diseases were more strongly associated with severe heatwaves than other health conditions. In contrast, there was an inverse association between heatwaves and cardiovascular admissions over lag 0–7 days. The heatwave–hospitalization associations were highest on the first day of exposure and declined thereafter for most hospitalization causes (S5 Fig). The exception was cardiovascular admissions, which were negatively associated with heatwaves over lag 0–7 days.

**Fig 4:**
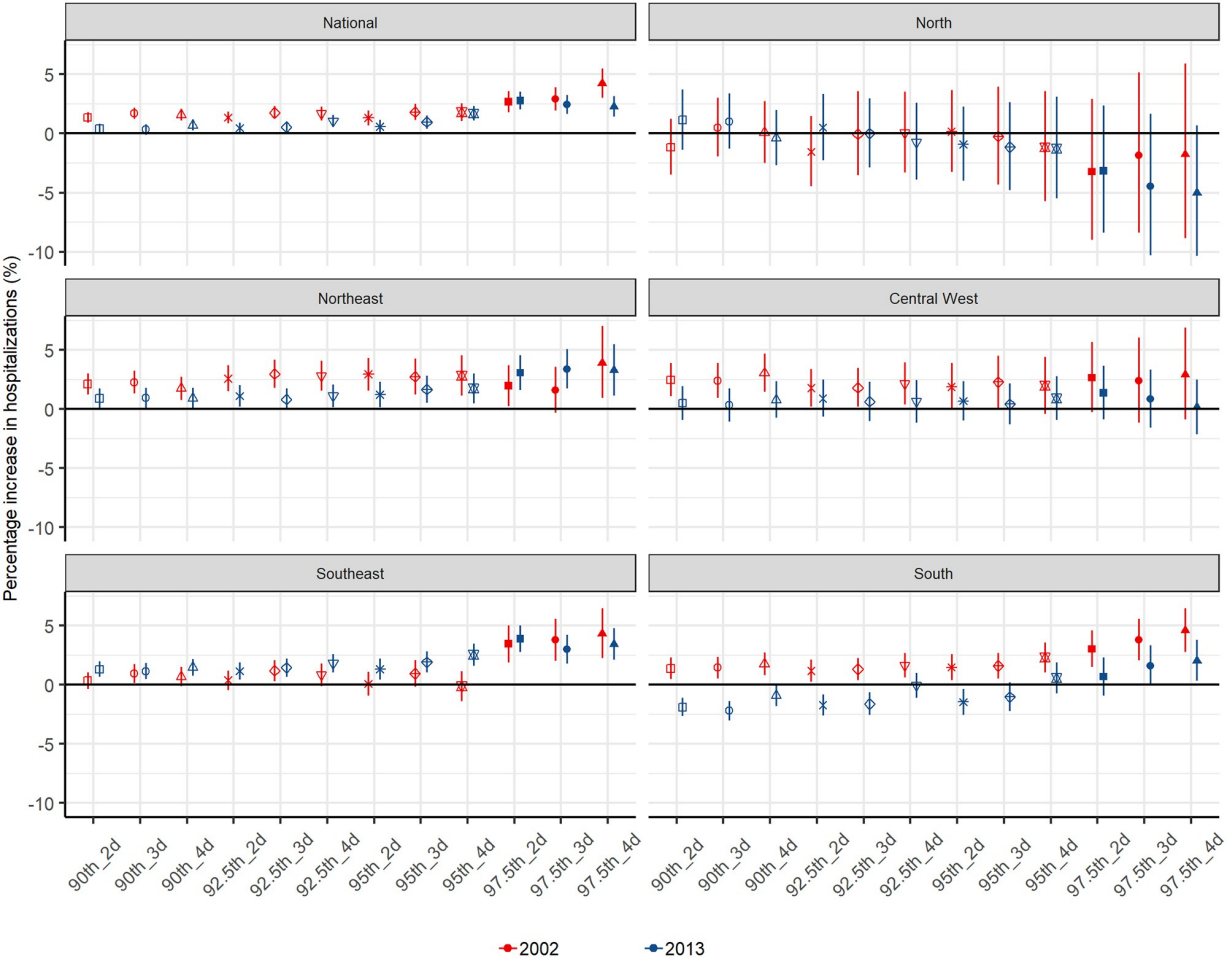
Associations between heatwaves (12 definitions) and risk of all-cause hospitalization by region at two time points. “2002” and “2013” indicate the middle days of the hottest five consecutive months in 2002 and 2013.

At the national level, the heatwave–hospitalization association declined temporally, especially for moderate heatwaves (Fig 4). Regionally, the association declined to the greatest extent in the south, with the association inverted for moderate heatwaves. Apparent declines were observed for most heatwaves in the central west and northeast. For the majority of heatwave definitions, there was an apparent increase in the heatwave–hospitalization association in the southeast.

Results of sensitivity analyses indicated that our results were robust to changed maximum lag days (7–9 days), df (3–5), and the inclusion of RH (S6–S8 Figs).

## Discussion

To the best of our knowledge, this is the first nationwide study to estimate the geographic, demographic, cause-specific, and temporal variations in the association between heatwaves and risk of hospitalization in Brazil. Using data covering more than 160 million residents, we observed that the risk of hospitalization—in particular for perinatal conditions—was higher during heatwaves with high temperatures and long durations than for moderate heatwaves. The nature of the association was complex and varied by health conditions, population demographics, and by region of the country. While there was no sex difference for heatwave susceptibility, children and the elderly were more vulnerable than adults. The association between heatwaves and risk of hospitalization was minimal in the north. In contrast, heatwaves with a threshold ≥97.5th percentile of year-round temperature showed excess health risk in the south and southeast after controlling for temperature. Over the study period, the risk of hospitalization associated with moderate heatwaves declined substantially at the national level, but regional differences existed.

The health risks associated with heatwaves have been increasingly reported over the past half century, with nearly all findings derived from Asia, Europe, North America, and Oceania [33]. In Vietnam, heatwaves have been associated with an up to 2.5% increase in hospitalization in 25 tropical cities [34]. A US study found that the risk of hospital admission in 114 cities increased by 1.6% to 3.8% during heatwave days [35]. Another study in Brisbane, Australia estimated heatwave-related admissions to be 5% to 18% higher compared with nonheatwave days [36]. Interestingly, the heatwave–hospitalization relationships observed in populations from Brazil and Vietnam are largely similar to those in high-income countries. This refutes past statements that populations in lower- and lower–middle-income countries are more susceptible to extreme heat due to limited health resources and poor infrastructure [1]. In agreement with this, a recent multicountry study that focused on heatwave-related mortality reported similar findings, with the authors speculating that heatwave adaptation may be greater in countries with hot climates such as Brazil than in cooler countries [24].

Previous studies have speculated that the relationship between heatwaves and health outcomes may be due to the inadequate response of the body’s thermoregulatory system [2,24,35]. However, the possible etiological pathways remain not fully understood. Our cause-specific results provide some insights regarding which physiological systems of the body may be susceptible to heatwaves. The highest risk of hospitalization occurred with perinatal conditions, which may be due to the immature nature of the physiological systems of newborns [37]. The strong association between heatwaves and hospitalizations for endocrine, nutritional and metabolic diseases, and skin problems were in agreement with clinical reporting of heat-related diseases, such as metabolic lactic acidosis and heat rash [38,39]. The dehydration and hypovolemia following exposure to extreme heat have been associated with renal failure and other genitourinary impairments [40]. There is evidence that heat stress may induce the release of inflammatory cytokines, causing the acute aggravation of respiratory disorders [41]. The associations between extreme high temperatures with injuries (e.g., violent crime and traffic accidents) and maternal conditions (e.g., preterm birth) have also been reported [42,43].

The adverse role of high temperature on the cardiovascular system has been well documented, including increases in heart rate and blood viscosity and declines in plasma volume and arterial blood pressure [44–46]. Paradoxically, we observed that the risk of cardiovascular admissions in Brazil declined after exposure to heatwaves. It is speculated that this may reflect a “harvesting effect” of extreme heat on individuals with pre-existing cardiovascular disease in the population, such that these individuals have a greater likelihood of dying before they are able to be hospitalized. Findings from a European study lend support to this hypothesis; in that study, heat exposure was inversely associated with cardiovascular morbidity but positively associated with cardiovascular mortality [41]. However, further studies are necessary to explore this phenomenon.

Population-level characteristics are important modifiers for the association between heatwaves and health outcomes within a country, with some studies suggesting that women may be more susceptible than men [47,48]. In contrast, our findings showed no substantial sex difference in the risk of hospitalization due to heat exposure. Brazils’ Unified Health System, which provides universally free access to health services for the Brazilian population, may largely explain this phenomenon [49]. Consistent with findings from other countries [50,51], children and the elderly in Brazil, particularly the population ≥70 years old, were more affected by heatwaves than adults. Both children and the elderly have lower thermoregulatory capacity compared with young and middle-aged adults [48]. In addition, there may be other explanatory variables, such as their lower awareness of drinking fresh water and electrolytes during extreme hot days.

Consistent with findings from countries with wide geographic areas [35], we also observed a regional variation in the association between heatwaves and hospitalizations within Brazil. The minimal association in the hottest region (the north) is in line with the hypothesis regarding adaptation; that is, people living in hot areas are less sensitive to heat exposure. However, the heatwave–hospitalization association in the coldest region (the south) was not significantly higher than regions with more moderate weather (the northeast and central west). This may be explained in part by the higher proportion of middle-aged adults—the population subgroup with the least heatwave susceptibility—in the south than other Brazilian areas [20]. The more advanced and balanced socioeconomic development in this area may also attenuate the population susceptibility to heatwaves [18].

In this study, the heatwave–hospitalization association diminished across most Brazilian regions after controlling for daily mean temperature. This is in line with some previous studies in other countries [24,35], indicating that the heatwave–hospitalization association may be largely represented by that of a single day’s high temperature for both morbidity and mortality. The exceptions were for the cold regions (the south and southeast), where the duration of extreme hot days associated with additional health risks. It remains unclear whether the added hospitalization risk related to the duration of severe heatwaves is associated with regional characteristics, which warrants investigation by further studies.

Numerous studies have reported a long-term decline in the strength of the association between extreme heat events and health outcomes in some of the most developed countries, such as the US and Japan, indicating adaptive capacity at the population level [14,52]. In Brazil, we also observed a reduced strength of heatwave–hospitalization association over time at the national level. This parallels Brazil’s economic development, infrastructure improvement (e.g., the generalization of air-conditioned and insulated buildings), and healthcare advancement in the past quarter century [12,53]. However, the attenuation was more apparent for heatwaves with low temperature thresholds, indicating that adaptation may be less effective against severe heatwaves. Our analysis shows a regional variation in this adaptive capacity, with the strongest adaptation occurring in the south of the country. Compared with other regions, the south has the most equitable distribution of socioeconomic indicators, such as the highest literacy rate [18,20]. The southeast region was the only area showing an apparent increase in heatwave susceptibility over the study period. This finding may be attributed to its having the greatest levels of socioeconomic inequality in the country, the greatest population density, and the highest proportion of people with poor health status [20,54].

IBGE estimates that the population of Brazil will remain relatively unchanged by 2060 [55]. However, the proportion of individuals ≥70 years will increase from 5% of the national population in 2015 to 19% in 2060, with the aging trend most severe in southern Brazil. Considering that there was minimal adaptation to heatwaves in populations in the southeast, the adverse association between heatwaves and risk of hospitalization—particularly among the elderly—should be considered. It is projected that the frequency, duration, and intensity of heatwaves will increase significantly over coming decades [56]. Given the lack of adaptability to severe heatwaves, the heatwave-related risk of hospitalization across the whole of the Brazilian population should also be considered in future healthcare planning and forecasting.

## Strengths and limitations

Our study has several strengths. First, this is the first nationwide study to explore the relationship between different definitions of heatwaves and risk of hospitalization across the Brazilian population and to quantify the temporal change. Our findings are relevant to policy makers and healthcare service providers because the results can inform future policy aimed at mitigating future impacts on healthcare utilization that are associated with sustained periods of high temperatures. Secondly, this study covers over three-quarters of the Brazilian population, suggesting that our results are broadly representative of the entire population of Brazil. At the global level, our study adds reliable and much-needed information to the sparse amount of knowledge that exists around the health risk associated with heatwaves.

This study also has several limitations. We used gridded temperature data rather than personal measurement. The measurement bias, if existent, should be randomly distributed across the Brazilian population, which may result in an underestimation of the heatwave–hospitalization association [57]. We were unable to adjust for air pollutants in the model because only a few Brazilian cities had long-term monitoring data during the 16-year study period. However, numerous studies have shown that the relationship between extreme heat and health outcomes is robust to the confounding effect of air pollutants [6,58]. We were also unable to provide mechanistic explanations regarding the association between heatwaves and a range of health outcomes due to the lack of more information at the individual level.

## Conclusions

In Brazil, heatwaves are associated with increased risk of hospitalization, with heatwaves characterized by high daily temperatures and long durations more vulnerable and less adaptable than moderate heatwaves. The heatwave–hospitalization association was unequally distributed but exhibited geographic, demographic, cause-specific, and temporal variations. Considering the predicted increase in the frequency, duration, and intensity of heatwaves under the current climate change scenarios, the health risk associated with heatwaves warrants further investigation. Adaptation strategies (e.g., developing a population-wide early warning system) and health promotion campaigns may help to reduce the burden of heatwave-related risk of hospitalization in Brazil and elsewhere in the world where heatwaves are becoming increasingly common.

## Supporting information

S1 Text STROBE Statement: Checklist of items that should be included in reports of observational studies. STROBE, Strengthening the Reporting of Observational Studies in Epidemiology.
(DOCX)

S2 Text Prospective analysis plan and modifications following comments from editors and reviewers. (DOCX)

S1 Table Causes of hospitalizations and ICD-10 codes. ICD-10, International Classification of Diseases, 10th revision.
(DOCX)

S2 Table Twelve heatwave definitions. (DOCX)

S3 Table Summary of the city-specific daily mean temperatures (°C, with standard deviations) across 1,814 Brazilian cities during 2000–2015. (DOCX)

S1 Fig Locations of 1,814 cities and the coverage (%) of local population in the five Brazilian regions. City-specific population sizes are extracted from Brazilian Census 2010 (http://www.censo2010.ibge.gov.br/).
(TIF)

S2 Fig Association between daily mean temperature and hospitalization (at the national level), modeled using a distributed lag nonlinear model. A natural cubic spline with 2 df was applied for daily temperature. df, degrees of freedom.
(TIF)

S3 Fig The heatwave–hospitalization association (90th_2d) across lag 0–7 days by region. (TIF)

S4 Fig The heatwave–hospitalization association (90th_2d) across lag 0–7 days by sex and age. (TIF)

S5 Fig The heatwave–hospitalization association (90th_2d) across lag 0–7 days by disease category. (TIF)

S6 Fig Results of sensitivity analyses by changing maximum lag days (7–9 days). (TIF)

S7 Fig Results of sensitivity analyses by changing df (3–5). df, degrees of freedom.
(TIF)

S8 Fig Results of sensitivity analyses by including RH. RH, relative humidity.
(TIF)

## Abbreviations

CI: confidence interval
df: degrees of freedom
IBGE: Brazil’s National Institute of Statistics
ICD-10: International Classification of Diseases, 10th revision
RH: relative humidity
STROBE: Strengthening the Reporting of Observational Studies in Epidemiology
