# Supplementary material for: The association between heatwaves and risk of hospitalization in Brazil: A nationwide time series study between 2000 and 2015
Source: PLoS Med. 2019 Feb 22;16(2):e1002753. doi: 10.1371/journal.pmed.1002753 (PMC6386221; doi:10.1371/journal.pmed.1002753)
Supplement: S1 Text — STROBE, Strengthening the Reporting of Observational Studies in Epidemiology. (DOCX) [file pmed.1002753.s001.docx]

**S1 Text. STROBE Statement — checklist of items that should be included in reports of observational studies**

|  | Item No | Recommendation | Relevant content in this paper |
| --- | --- | --- | --- |
| **Title and abstract** | 1 | (*a*) Indicate the study’s design with a commonly used term in the title or the abstract | In Title: ‘time series study’ |
|  |  | (*b*) Provide in the abstract an informative and balanced summary of what was done and what was found | In Methods and Findings section, Abstract. |
| Introduction | | |  |
| Background/rationale | 2 | Explain the scientific background and rationale for the investigation being reported | In para 1-3, Introduction |
| Objectives | 3 | State specific objectives, including any prespecified hypotheses | In para 4, Introduction |
| Methods | | |  |
| Study design | 4 | Present key elements of study design early in the paper | In para 1, Methods: ‘time series study’. Details of study design are presented in the Statistical analyses section, Methods. |
| Setting | 5 | Describe the setting, locations, and relevant dates, including periods of recruitment, exposure, follow-up, and data collection | In Data collection section, Methods: ‘For each city, time series data on hospitalization between 1 January 2000 and 31 December 2015 were collected through Brazil’s National Unified Health System.’ |
| Participants | 6 | (*a*) *Cohort study*—Give the eligibility criteria, and the sources and methods of selection of participants. Describe methods of follow-up  *Case-control study*—Give the eligibility criteria, and the sources and methods of case ascertainment and control selection. Give the rationale for the choice of cases and controls  *Cross-sectional study*—Give the eligibility criteria, and the sources and methods of selection of participants | In Data collection section, Methods: ‘There were 5,570 cities in Brazil at the end of 2015, but the electronic medical records of some cities were not completed during the early years. To reduce the impact of missing data, we only applied for hospitalization records in 1,814 cities that had complete data for the 16-year study period.’ |
|  |  | (*b*) *Cohort study*—For matched studies, give matching criteria and number of exposed and unexposed  *Case-control study*—For matched studies, give matching criteria and the number of controls per case | NA  NA |
| Variables | 7 | Clearly define all outcomes, exposures, predictors, potential confounders, and effect modifiers. Give diagnostic criteria, if applicable | These are defined and presented in the Data collection section, Definitions of heatwaves section and Statistical analyses section, Methods. **Outcomes** are the city-specific daily counts of hospitalizations, which are divided into sex (men and women), 10 age groups (0−4, 5−9, 10−19, 20−29, 30−39, 40−49, 50−59, 60−69, 70−79, or ≥80 years), and nine cause categories (S1 Table). **Exposures**, i.e. heatwaves are: ‘In this study, we applied 12 heatwave definitions (S2 Table), by combining thresholds at the 90th, 92.5th, 95th or 97.5th percentiles of city-specific year-round daily mean temperatures and durations ≥ 2, 3 or 4 consecutive days, respectively.’ **Covariates** are presented in Eq 1, including a categorical variable combining the year and calendar month, a categorical variable representing day of the week, and a binary variable representing public holidays. |
| Data sources/ measurement | 8* | For each variable of interest, give sources of data and details of methods of assessment (measurement). Describe comparability of assessment methods if there is more than one group | These are presented in the Data collection section, Methods. **Hospitalization variables**: ‘For each city, time series data on hospitalization between 1 January 2000 and 31 December 2015 were collected through Brazil’s National Unified Health System.’ and ‘Medical variables included information on patient’s sex, age (0−4, 5−9, 10−19, 20−29, 30−39, 40−49, 50−59, 60−69, 70−79, or ≥80 years), admission date, city of residence, and primary diagnosis coded according to the International Classification of Diseases, 10th revision (ICD-10). The hospitalization data were then divided into nine main cause categories according to primary diagnosis (S1 Table)’ **Temperature variables** (used to define heatwaves): ‘For each city, daily minimum and maximum temperatures were extracted from a 0.25° × 0.25° meteorological dataset’ and ‘data from the grid overlaying the center of each city were applied. Daily mean temperature was computed as the mean of minimum and maximum temperatures.’ |
| Bias | 9 | Describe any efforts to address potential sources of bias | These are presented in the Sensitivity analyses section, Methods. |
| Study size | 10 | Explain how the study size was arrived at | In Data collection section, Methods: ‘For each city, time series data on hospitalization between 1 January 2000 and 31 December 2015 were collected through Brazil’s National Unified Health System. There were 5,570 cities in Brazil at the end of 2015, but the electronic medical records of some cities were not completed during the early years. To reduce the impact of missing data, we only applied for hospitalization records in 1,814 cities that had complete data for the 16-year study period.’ |
| Quantitative variables | 11 | Explain how quantitative variables were handled in the analyses. If applicable, describe which groupings were chosen and why | These are presented in the Data collection section, Methods. Ages of patients are divided into 10 groups (0−4, 5−9, 10−19, 20−29, 30−39, 40−49, 50−59, 60−69, 70−79, or ≥80 years). |
| Statistical methods | 12 | (*a*) Describe all statistical methods, including those used to control for confounding | In Statistical analyses section, Methods. |
|  |  | (*b*) Describe any methods used to examine subgroups and interactions | In Statistical analyses section, Methods |
|  |  | (*c*) Explain how missing data were addressed | Analyses were based on complete hospitalization records in the 16-year study period. |
|  |  | (*d*) *Cohort study*—If applicable, explain how loss to follow-up was addressed  *Case-control study*—If applicable, explain how matching of cases and controls was addressed  *Cross-sectional study*—If applicable, describe analytical methods taking account of sampling strategy | NA |
|  |  | (*e*) Describe any sensitivity analyses | These are presented in the Sensitivity analyses section, Methods. |

| Results | | |  |
| --- | --- | --- | --- |
| Participants | 13* | (a) Report numbers of individuals at each stage of study—eg numbers potentially eligible, examined for eligibility, confirmed eligible, included in the study, completing follow-up, and analysed | In para 1, Results |
|  |  | (b) Give reasons for non-participation at each stage | NA |
|  |  | (c) Consider use of a flow diagram | NA |
| Descriptive data | 14* | (a) Give characteristics of study participants (eg demographic, clinical, social) and information on exposures and potential confounders | In para 1, Results |
|  |  | (b) Indicate number of participants with missing data for each variable of interest | NA |
|  |  | (c) *Cohort study*—Summarise follow-up time (eg, average and total amount) | NA |
| Outcome data | 15* | *Cohort study*—Report numbers of outcome events or summary measures over time | NA |
|  |  | *Case-control study—*Report numbers in each exposure category, or summary measures of exposure | NA |
|  |  | *Cross-sectional study—*Report numbers of outcome events or summary measures | In para 1, Results |
| Main results | 16 | (*a*) Give unadjusted estimates and, if applicable, confounder-adjusted estimates and their precision (eg, 95% confidence interval). Make clear which confounders were adjusted for and why they were included | In para 2, Results |
|  |  | (*b*) Report category boundaries when continuous variables were categorized | In para 3, Results and Fig 2 for age-specific results |
|  |  | (*c*) If relevant, consider translating estimates of relative risk into absolute risk for a meaningful time period | NA |
| Other analyses | 17 | Report other analyses done—eg analyses of subgroups and interactions, and sensitivity analyses | In para 3-6, Results |
| Discussion | | |  |
| Key results | 18 | Summarise key results with reference to study objectives | In para 1, Discussion |
| Limitations | 19 | Discuss limitations of the study, taking into account sources of potential bias or imprecision. Discuss both direction and magnitude of any potential bias | In Strengths & Limitations |
| Interpretation | 20 | Give a cautious overall interpretation of results considering objectives, limitations, multiplicity of analyses, results from similar studies, and other relevant evidence | In para 2-8, Discussion |
| Generalisability | 21 | Discuss the generalisability (external validity) of the study results | In para 9, Discussion and Strengths & Limitations |
| Other information | | |  |
| Funding | 22 | Give the source of funding and the role of the funders for the present study and, if applicable, for the original study on which the present article is based | In Funding |

*Give information separately for cases and controls in case-control studies and, if applicable, for exposed and unexposed groups in cohort and cross-sectional studies.

**Note:** An Explanation and Elaboration article discusses each checklist item and gives methodological background and published examples of transparent reporting. The STROBE checklist is best used in conjunction with this article (freely available on the Web sites of PLoS Medicine at http://www.plosmedicine.org/, Annals of Internal Medicine at http://www.annals.org/, and Epidemiology at http://www.epidem.com/). Information on the STROBE Initiative is available at www.strobe-statement.org.
