# Supplementary material for: The association between heatwaves and risk of hospitalization in Brazil: A nationwide time series study between 2000 and 2015
Source: PLoS Med. 2019 Feb 22;16(2):e1002753. doi: 10.1371/journal.pmed.1002753 (PMC6386221; doi:10.1371/journal.pmed.1002753)
Supplement: S2 Text — (DOCX) [file pmed.1002753.s002.docx]

**S2 Text. Prospective analysis plan and modifications following comments from editors and reviewers**

**Date of commencing this research:** March 20, 2018.

**Research type:** A time series study

**Research objectives:**

1. To quantify the associations between different heatwave definitions and hospitalizations in Brazil between 2000 and 2015.

2. To explore whether the heatwave-hospitalization associations varied across Brazilian five regions, population subgroups (i.e. sex and age), and nine cause categories (S2 Table).

3. To explore whether the heatwave-hospitalization associations varied temporally over the 16-year study period.

**Subjects:**

Daily hospitalization cases from 1,814 Brazilian cities in the hottest five consecutive months during 2000−2015. Data were extracted from the Brazil’s National Unified Health System.

**Exposure measurements:**

For each city, 12 heatwave definitions will be applied by combining thresholds at the 90th, 92.5th, 95th or 97.5th percentiles of year-round city-specific daily mean temperatures and durations ≥ 2, 3 or 4 consecutive days, respectively.

**Outcomes:**

For each heatwave definition, the percentage change in the risk of hospitalization during heatwave days (with 95% confidence interval), compared with non-heatwave days.

**Analyses:**

1. Descriptive analyses

Basic characteristics of hospitalizations and heatwaves in Brazil during the study period will be summarized, such as the number/rate of hospitalizations, the sex and age distributions of hospitalizations, and the number of days of different heatwave definitions.

2. Statistical analyses

1) Heatwave-hospitalization associations

A two-stage approach will be performed. First, a quasi-Poisson regression with constrained distributed lag model will be fitted to obtain city-specific estimates with controlling for confounding factors (i.e., long-term trend, intra-seasonal variation, day of the week and public holidays). Second, city-specific estimates will be pooled at the national and regional levels using a random-effect meta-analysis with maximum likelihood estimation. Stratified analyses will be performed by sex (men and women), 10 age groups (0−4, 5−9, 10−19, 20−29, 30−39, 40−49, 50−59, 60−69, 70−79, and ≥80 years), and nine cause categories (S2 Table).

2) Long-term change in the associations

A two-stage strategy will be performed. First, the city-specific estimates per year will be extracted. Second, meta-regression will be performed to estimate the annual change in the association at national and regional levels.

3. Additional analyses

Sensitivity analyses will be performed to test the robustness of our findings.

**Modification based on the comments from editors and reviewers:**

Following the suggestions of reviewers, we examined the added effect of heatwaves by adjusting for daily mean temperature in the first-stage model. The long-term changes in the heatwave-hospitalization associations were re-performed using quasi-Poisson regression with time-varying constrained distributed lag model.
