# Supplementary material for: The association between heatwaves and risk of hospitalization in Brazil: A nationwide time series study between 2000 and 2015
Source: PLoS Med. 2019 Feb 22;16(2):e1002753. doi: 10.1371/journal.pmed.1002753 (PMC6386221; doi:10.1371/journal.pmed.1002753)
Supplement: S1 Table — ICD-10, International Classification of Diseases, 10th revision. (DOCX) [file pmed.1002753.s003.docx]

**S1 Table. Causes of hospitalizations and ICD-10 codes.**

| **No.** | **Causes** | **ICD-10 codes** |
| --- | --- | --- |
| 1 | Neoplasms | C00-D48 |
| 2 | Endocrine, nutritional and metabolic diseases | E00-E90 |
| 3 | Diseases of the cardiovascular system | I00-I99 |
| 4 | Diseases of the respiratory system | J00-J99 |

| 5 | Diseases of the skin and subcutaneous tissue | L00-L99 |
| --- | --- | --- |

| 6 | Diseases of the genitourinary system | N00-N99 |
| --- | --- | --- |
| 7 | Maternal conditions | O00-O99 |
| 8 | Certain conditions originating in the perinatal period | P00-P99 |
| 9 | Injury, poisoning and certain other consequences of external causes | S00-T98 |
