# Supplementary material for: The association between heatwaves and risk of hospitalization in Brazil: A nationwide time series study between 2000 and 2015
Source: PLoS Med. 2019 Feb 22;16(2):e1002753. doi: 10.1371/journal.pmed.1002753 (PMC6386221; doi:10.1371/journal.pmed.1002753)
Supplement: S2 Table — (DOCX) [file pmed.1002753.s004.docx]

**S2 Table. Twelve heatwave definitions.**

| **No.** | **Threshold (%)** | **Duration (days)** | **Abbreviation of heatwave definition** |
| --- | --- | --- | --- |
| 1 | 90th | 2 | 90th_2d |
| 2 | 90th | 3 | 90th_3d |
| 3 | 90th | 4 | 90th_4d |
| 4 | 92.5th | 2 | 92.5th_2d |
| 5 | 92.5th | 3 | 92.5th_3d |
| 6 | 92.5th | 4 | 92.5th_4d |
| 7 | 95th | 2 | 95th_2d |
| 8 | 95th | 3 | 95th_3d |
| 9 | 95th | 4 | 95th_4d |
| 10 | 97.5th | 2 | 97.5th_2d |
| 11 | 97.5th | 3 | 97.5th_3d |
| 12 | 97.5th | 4 | 97.5th_4d |
