# Supplementary material for: The association between heatwaves and risk of hospitalization in Brazil: A nationwide time series study between 2000 and 2015
Source: PLoS Med. 2019 Feb 22;16(2):e1002753. doi: 10.1371/journal.pmed.1002753 (PMC6386221; doi:10.1371/journal.pmed.1002753)
Supplement: S3 Table — (DOCX) [file pmed.1002753.s005.docx]

**S3 Table. Summary of the city-specific daily mean temperatures (℃, ±SD) across 1,814 Brazilian cities during 2000−2015.**

| **Regions** | **Mean** | **90th** | **92.5th** | **95th** | **97.5th** |
| --- | --- | --- | --- | --- | --- |
| National | 23.5 ± 2.8 | 26.8 ± 1.8 | 27.1 ± 1.7 | 27.5 ± 1.7 | 28.0 ± 1.6 |
| North | 27.1 ± 0.7 | 28.7 ± 0.6 | 28.9 ± 0.6 | 29.1 ± 0.6 | 29.5 ± 0.6 |
| Northeast | 26.0 ± 1.6 | 28.2 ± 1.4 | 28.4 ± 1.3 | 28.7 ± 1.3 | 29.1 ± 1.3 |
| Central west | 25.0 ± 1.3 | 27.7 ± 1.2 | 28.0 ± 1.2 | 28.4 ± 1.2 | 29.0 ± 1.2 |
| Southeast | 22.3 ± 1.5 | 25.9 ± 1.3 | 26.2 ± 1.3 | 26.6 ± 1.3 | 27.2 ± 1.3 |
| South | 20.2 ± 1.5 | 25.3 ± 1.2 | 25.7 ± 1.2 | 26.2 ± 1.1 | 27.0 ± 1.1 |
