# Supplementary figures and images for: The association between heatwaves and risk of hospitalization in Brazil: A nationwide time series study between 2000 and 2015
Source: PLoS Med. 2019 Feb 22;16(2):e1002753. doi: 10.1371/journal.pmed.1002753 (PMC6386221; doi:10.1371/journal.pmed.1002753)

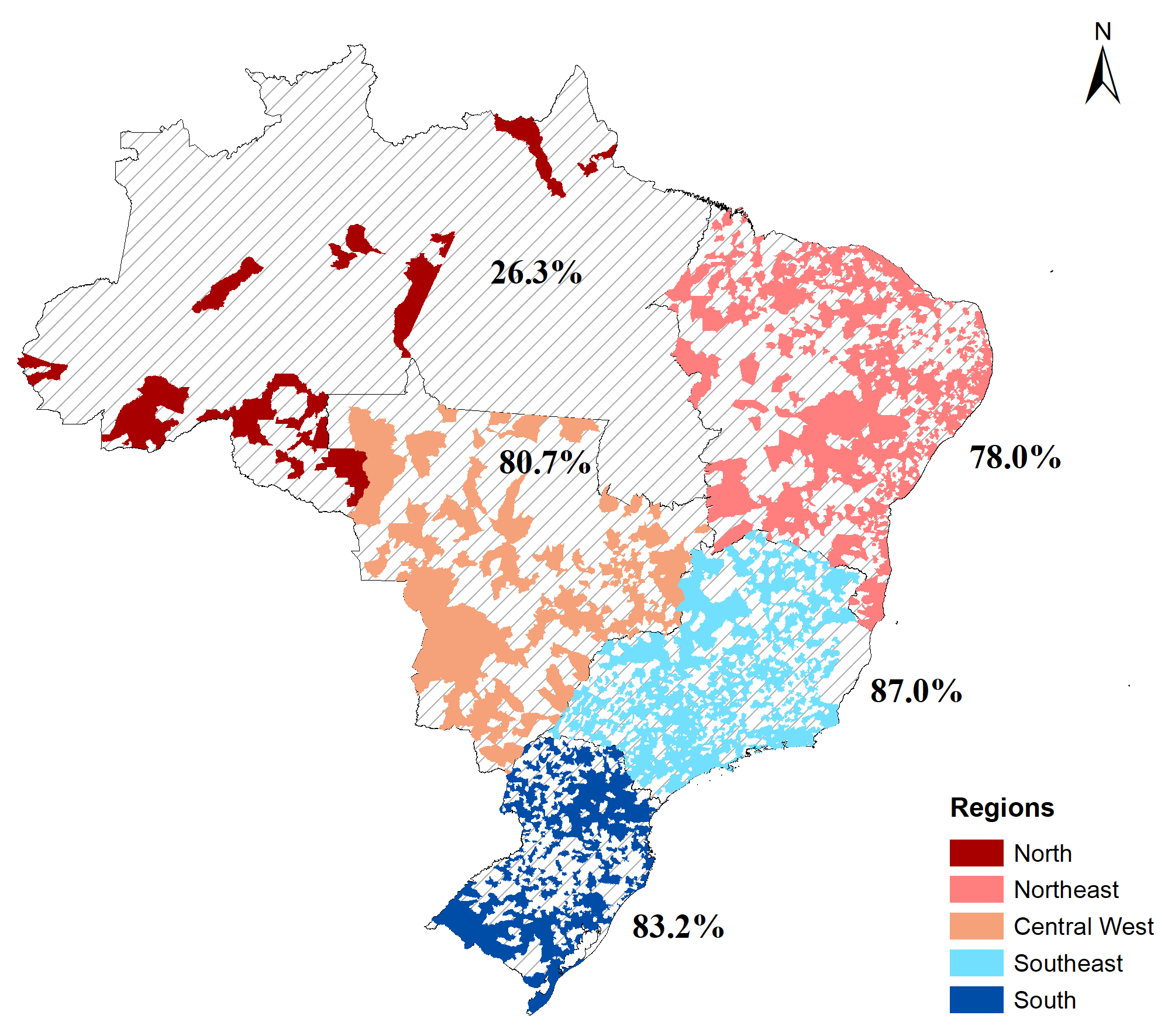

Supplement: S1 Fig — City-specific population sizes are extracted from Brazilian Census 2010 (http://www.censo2010.ibge.gov.br/). (TIF) [file pmed.1002753.s006.tif]

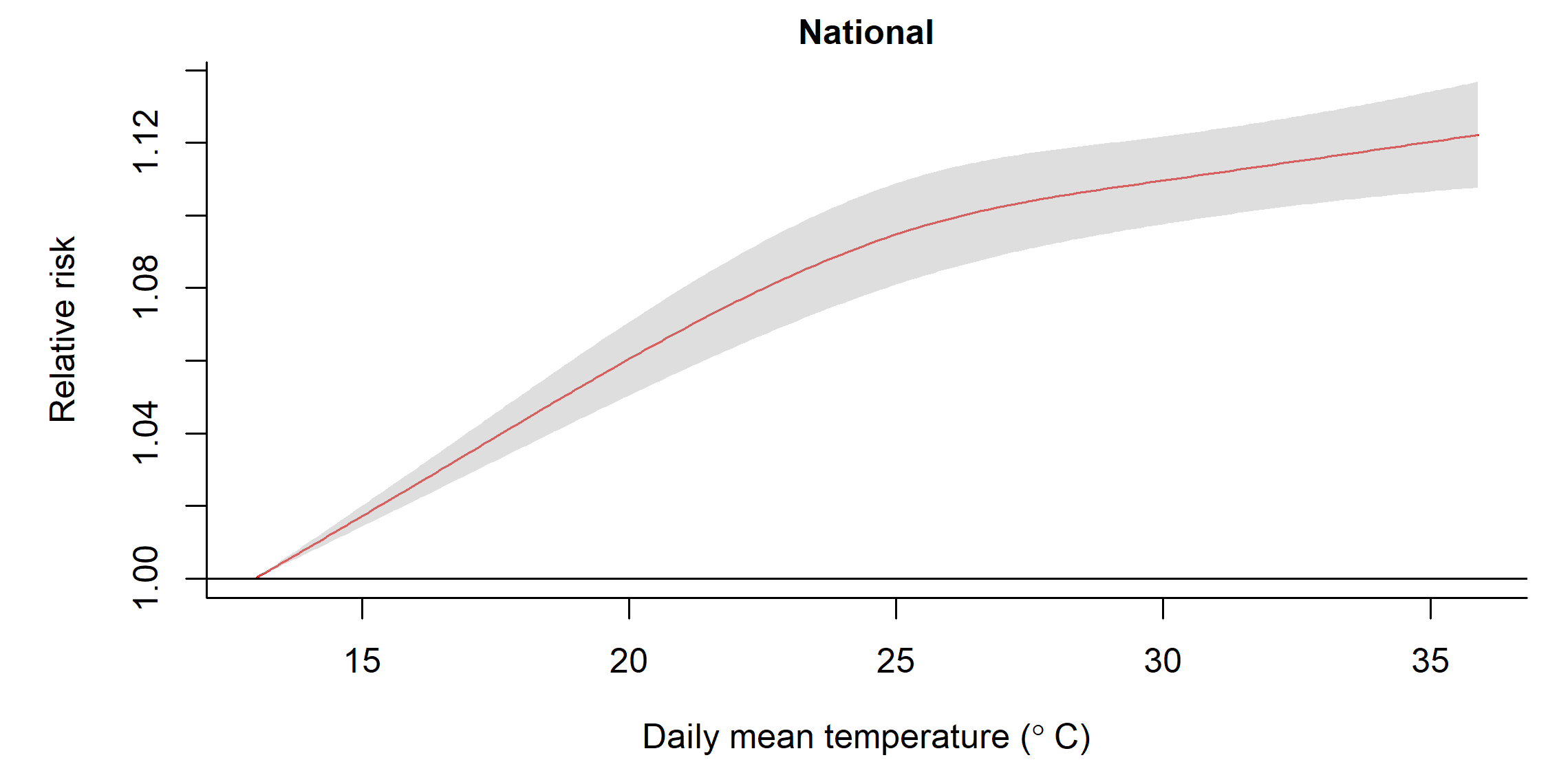

Supplement: S2 Fig — A natural cubic spline with 2 df was applied for daily temperature. df, degrees of freedom. (TIF) [file pmed.1002753.s007.tif]

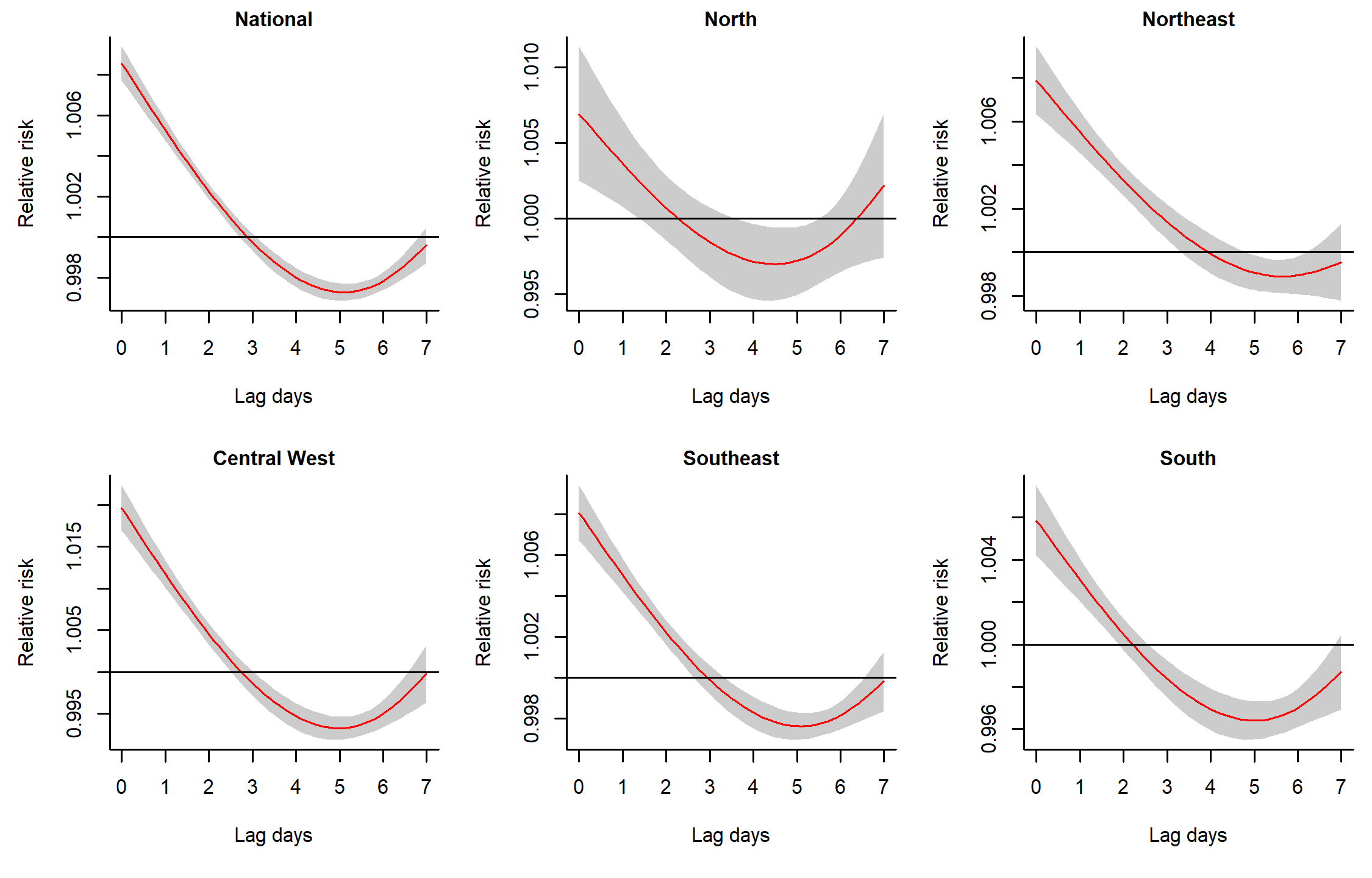

Supplement: S3 Fig — (TIF) [file pmed.1002753.s008.tif]

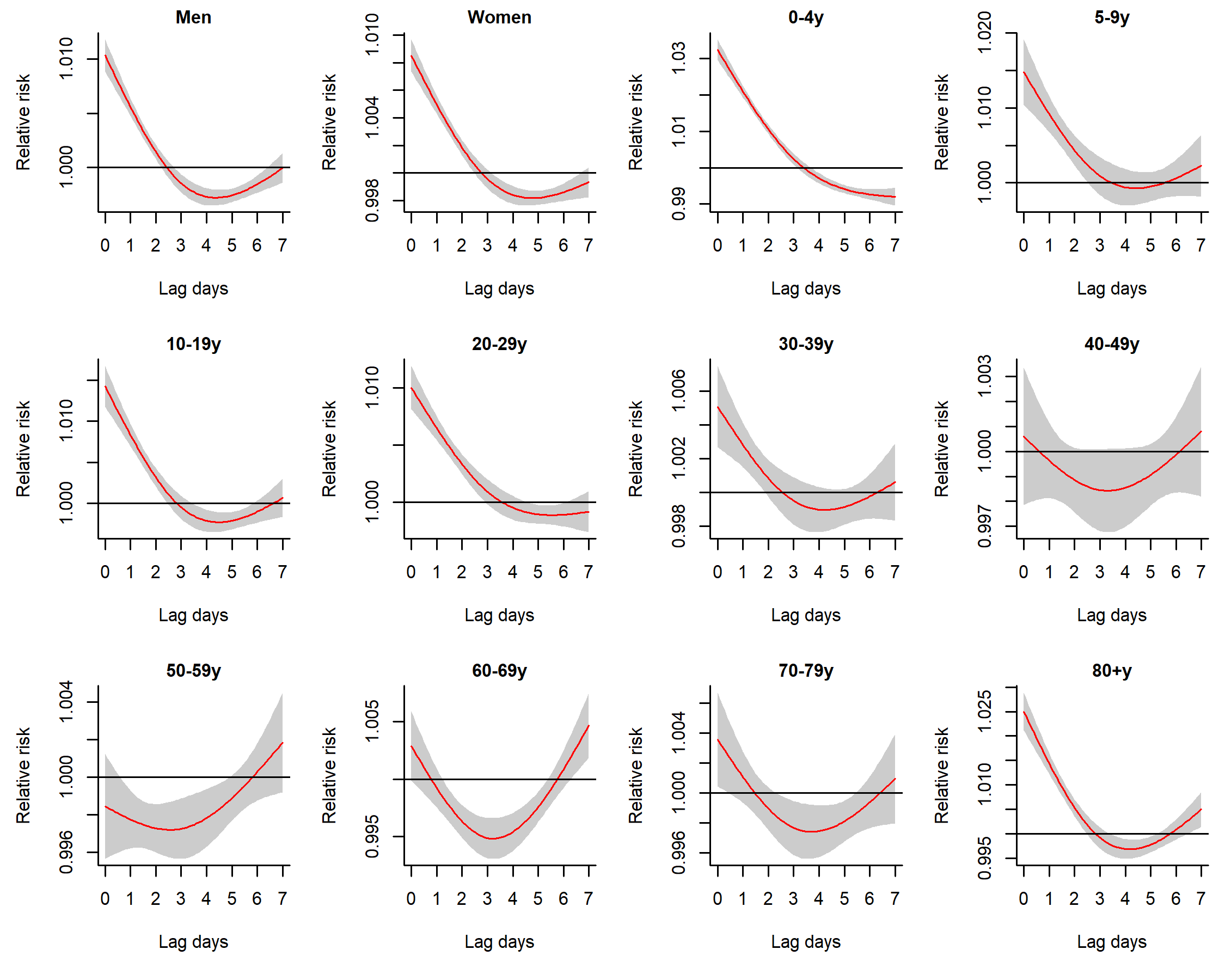

Supplement: S4 Fig — (TIF) [file pmed.1002753.s009.tif]

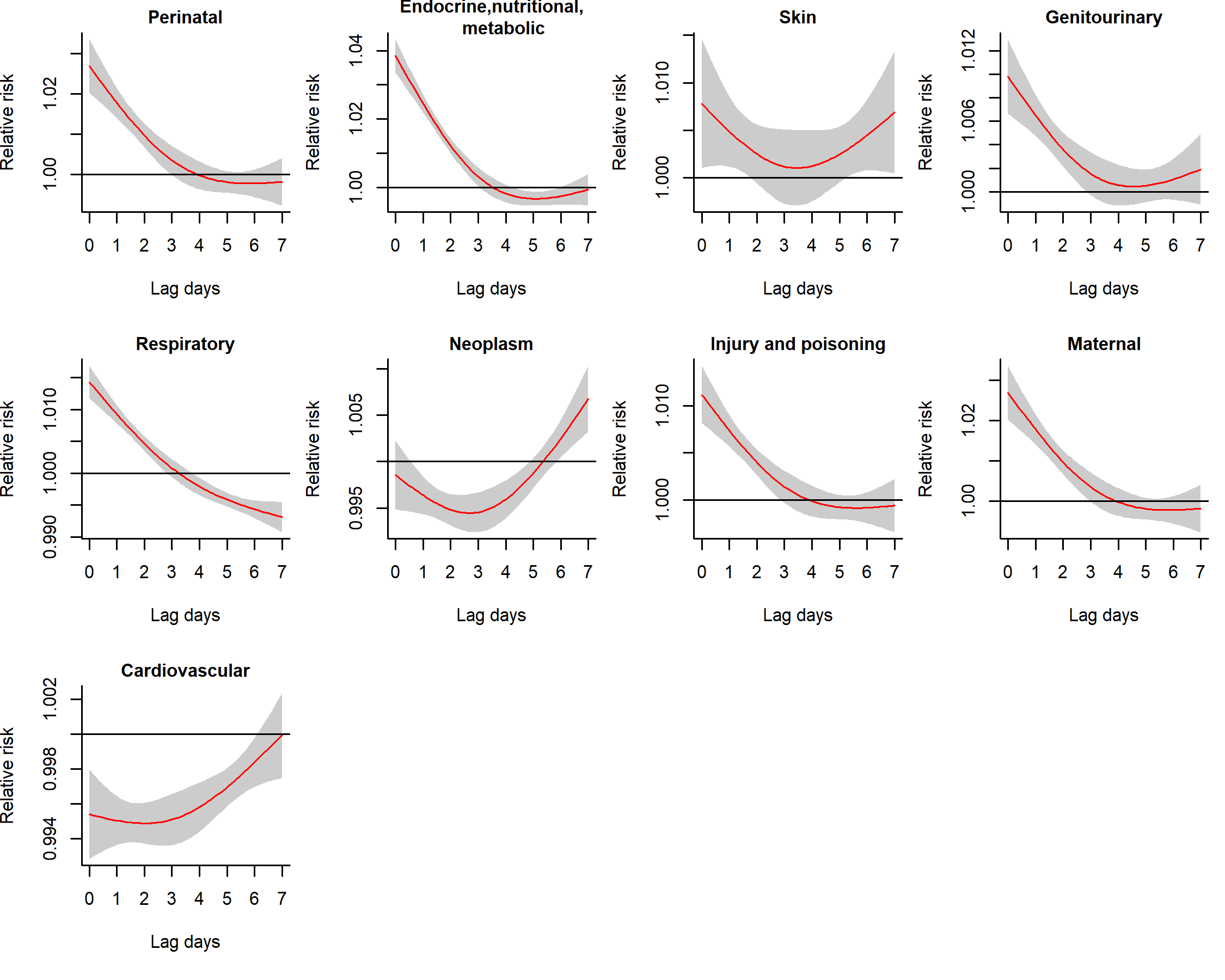

Supplement: S5 Fig — (TIF) [file pmed.1002753.s010.tif]

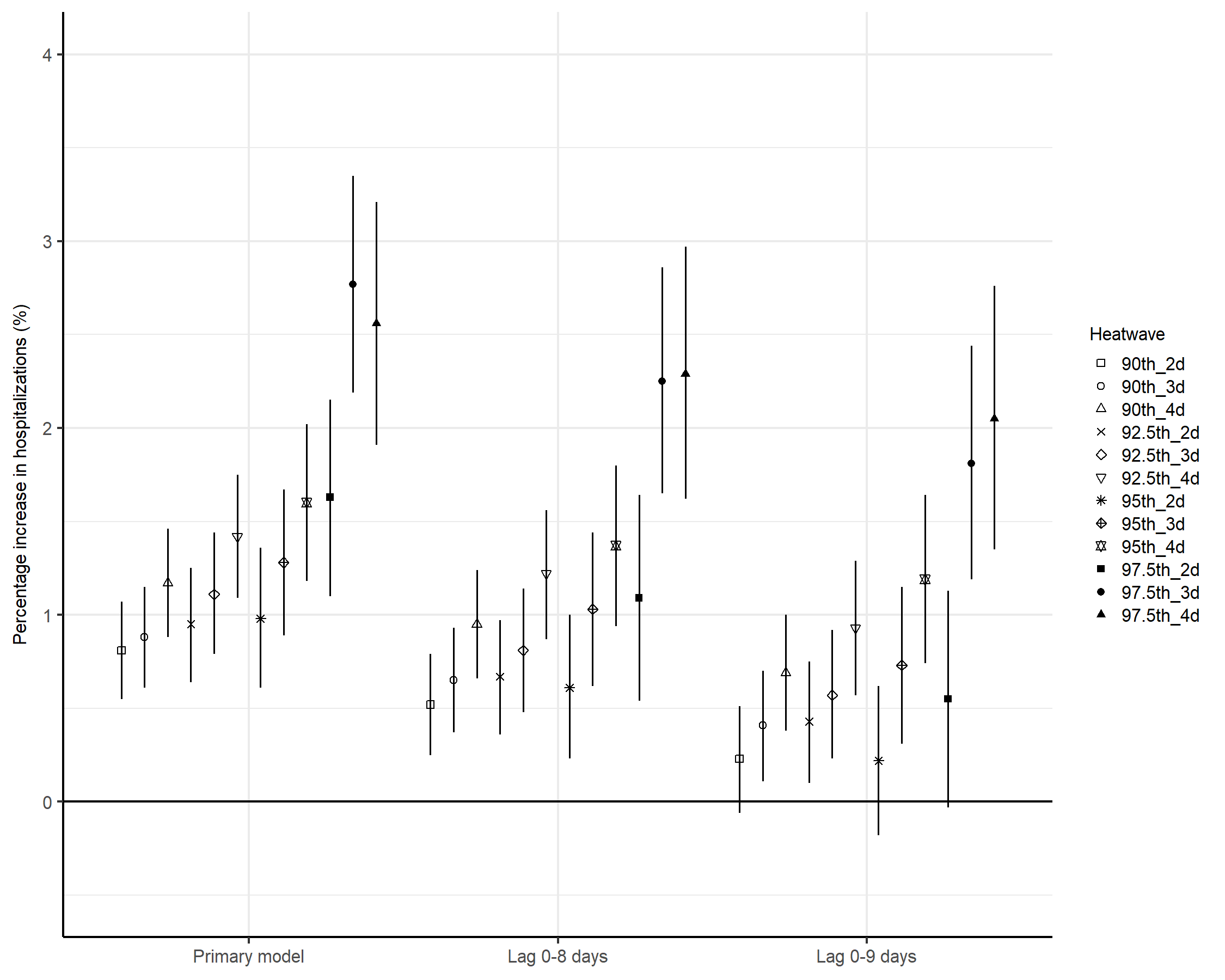

Supplement: S6 Fig — (TIF) [file pmed.1002753.s011.tif]

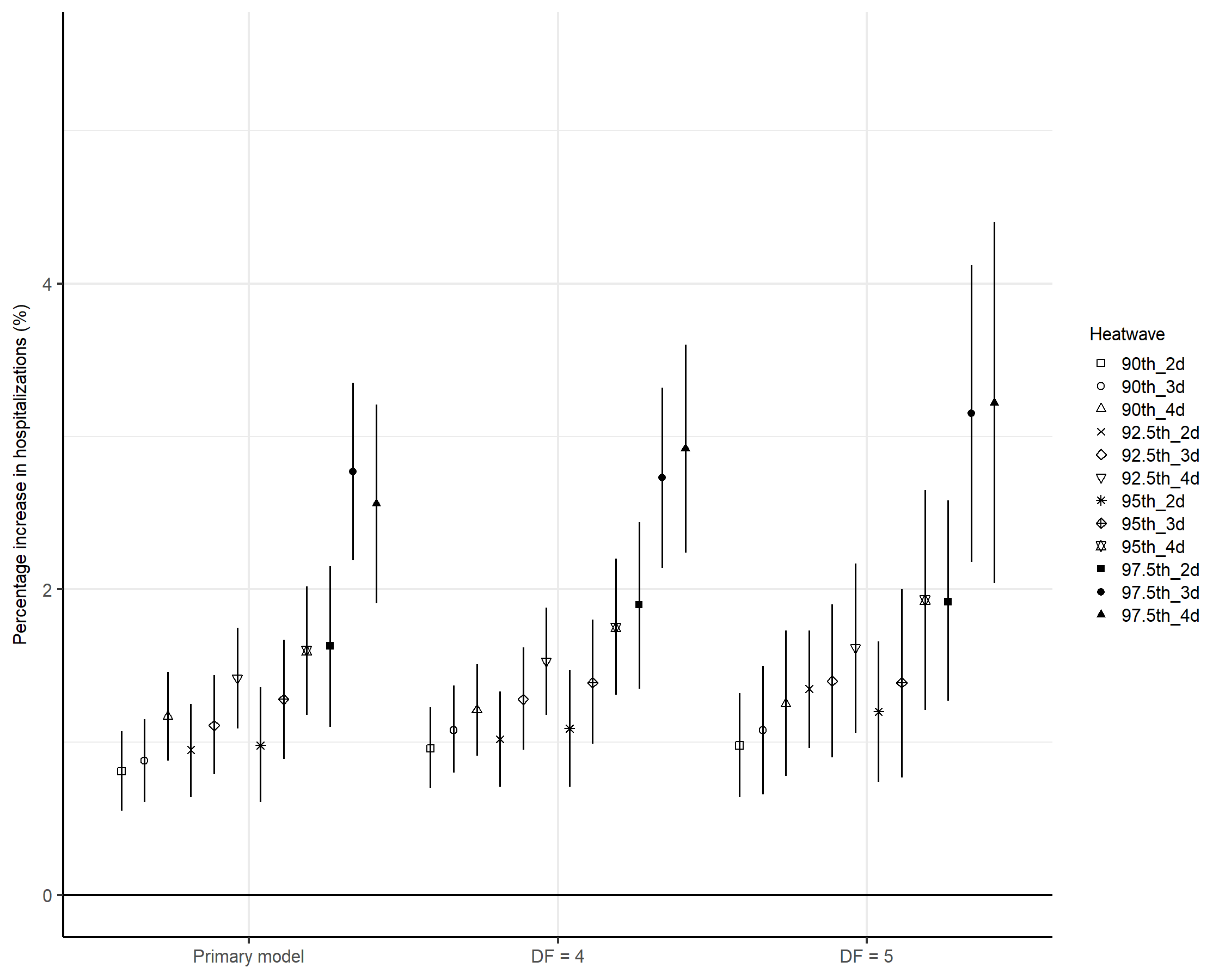

Supplement: S7 Fig — df, degrees of freedom. (TIF) [file pmed.1002753.s012.tif]

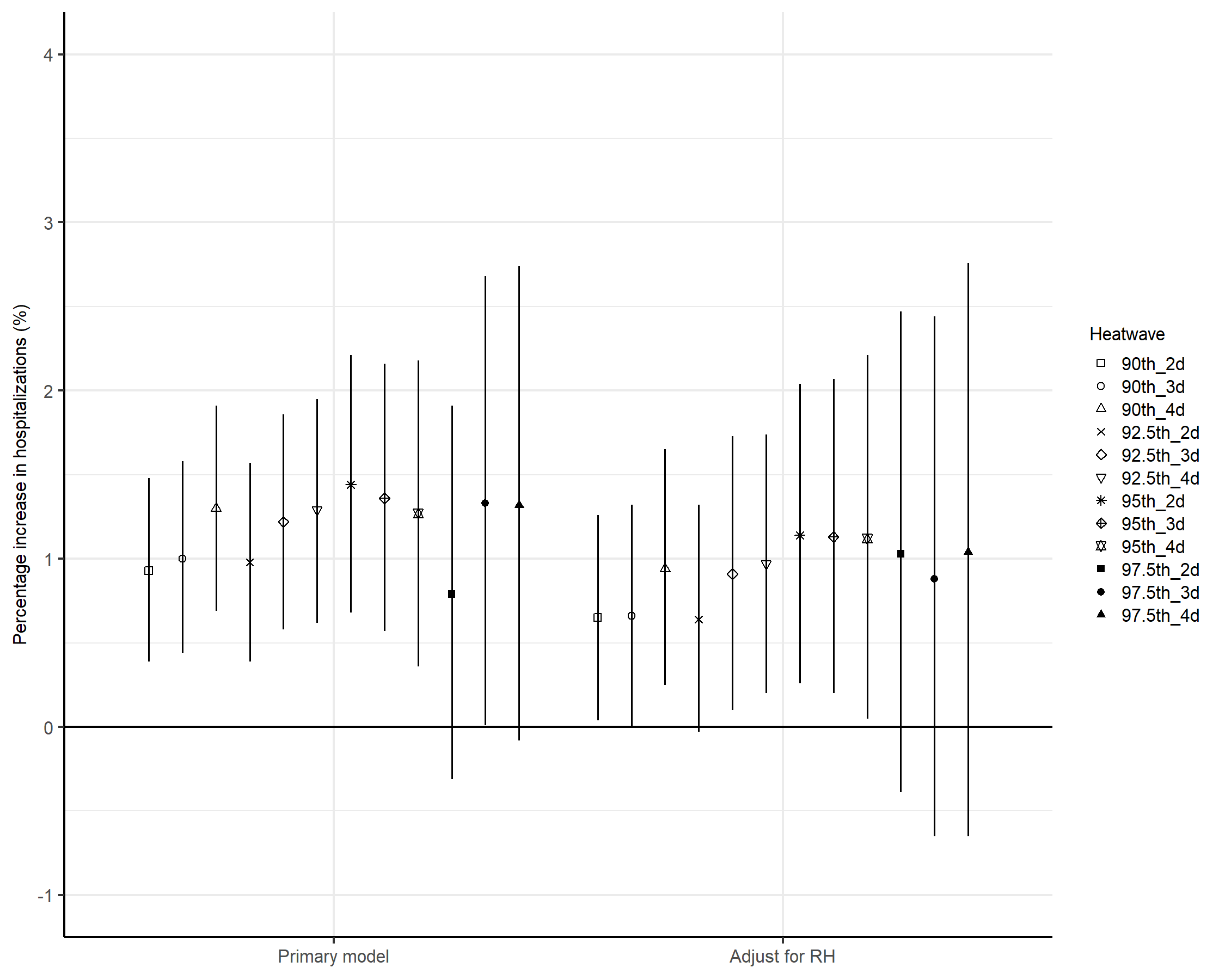

Supplement: S8 Fig — RH, relative humidity. (TIF) [file pmed.1002753.s013.tif]
